# Supplementary figures and images for: Neutrophils Contribute to ER Stress in Lung Epithelial Cells in the Pristane-Induced Diffuse Alveolar Hemorrhage Mouse Model
Source: Front Immunol. 2022 Feb 3;13:790043. doi: 10.3389/fimmu.2022.790043 (PMC8850275; doi:10.3389/fimmu.2022.790043)

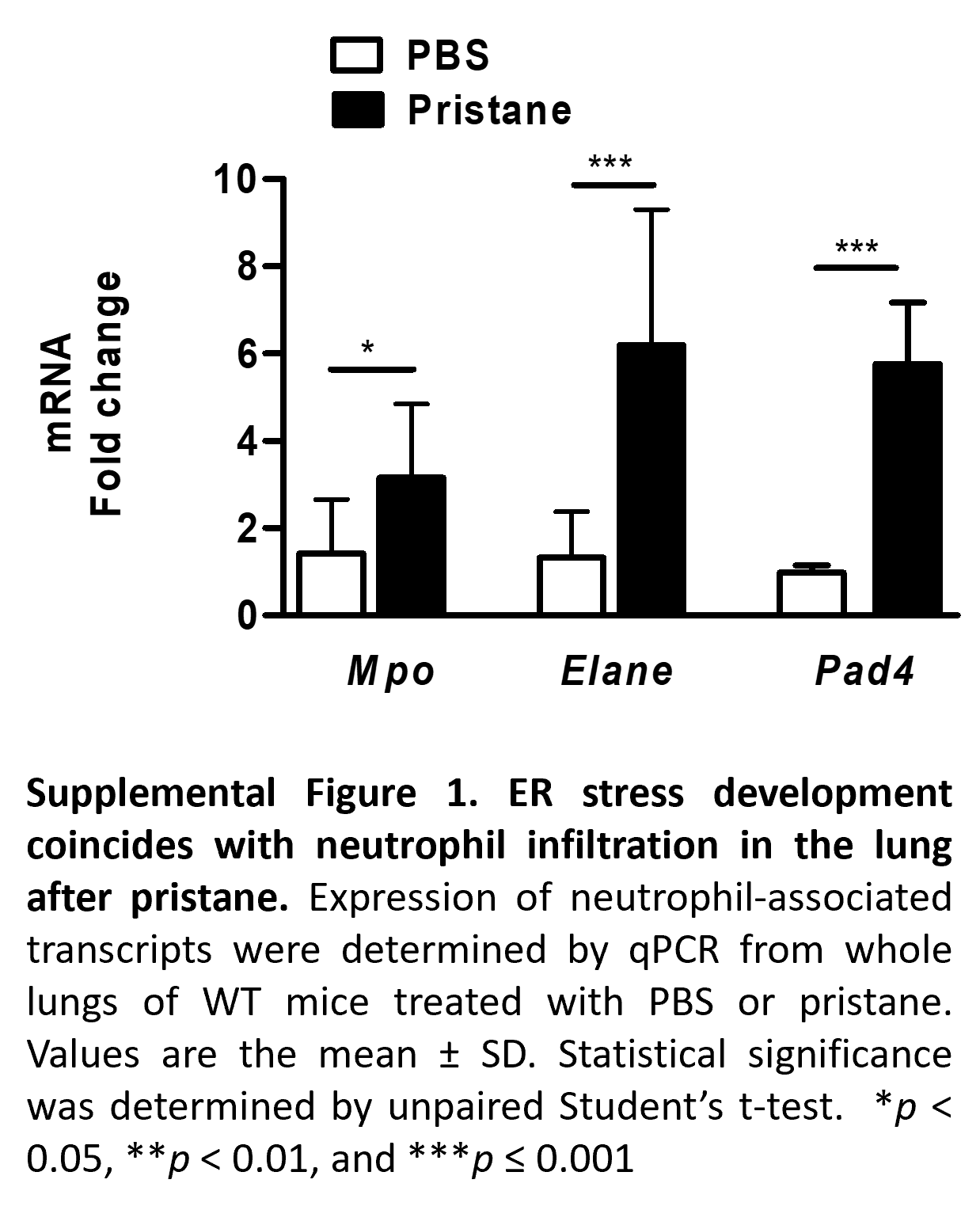

Supplement: Supplementary file 1 [file Image_1.jpg]

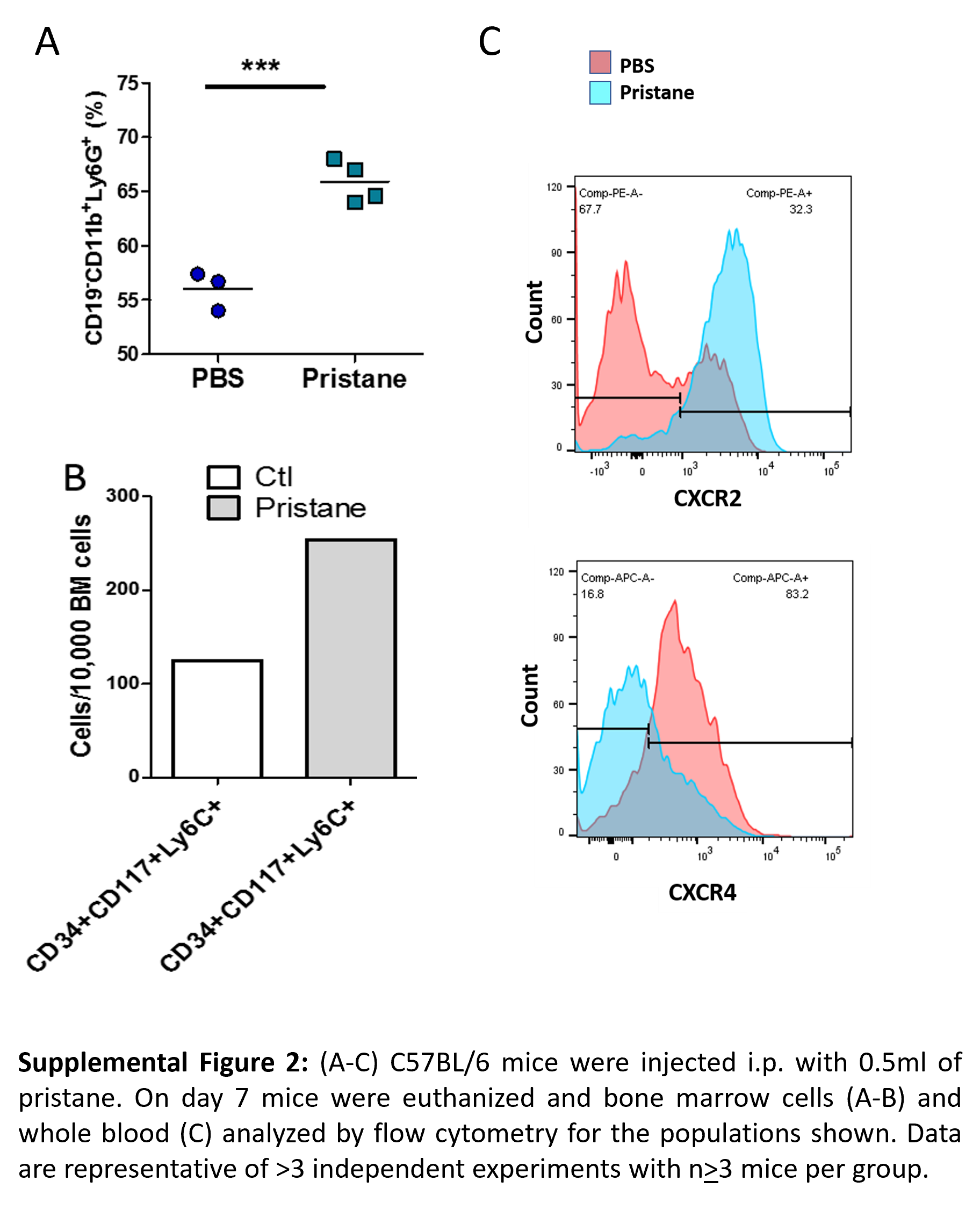

Supplement: Supplementary file 2 [file Image_2.jpg]

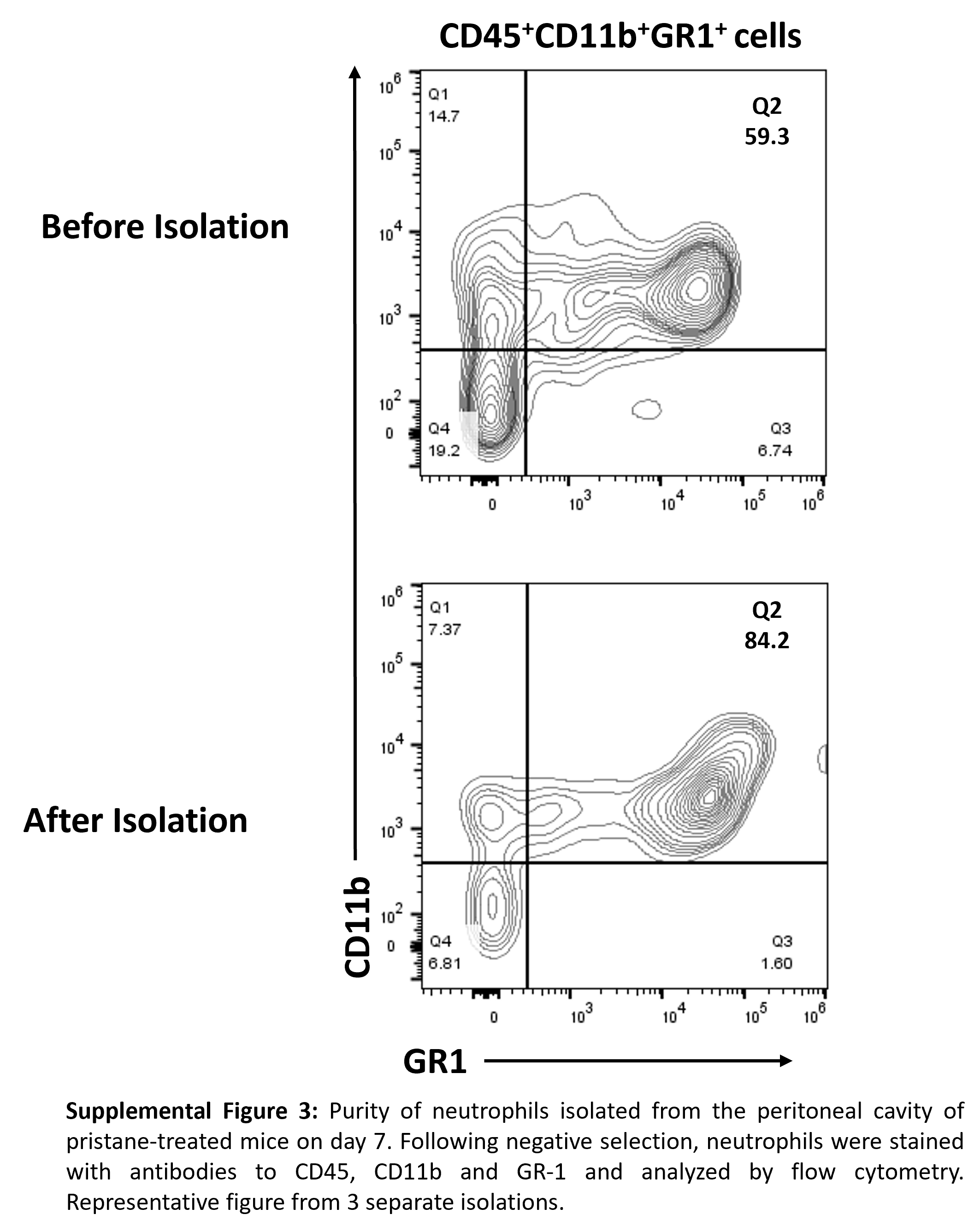

Supplement: Supplementary file 3 [file Image_3.jpg]

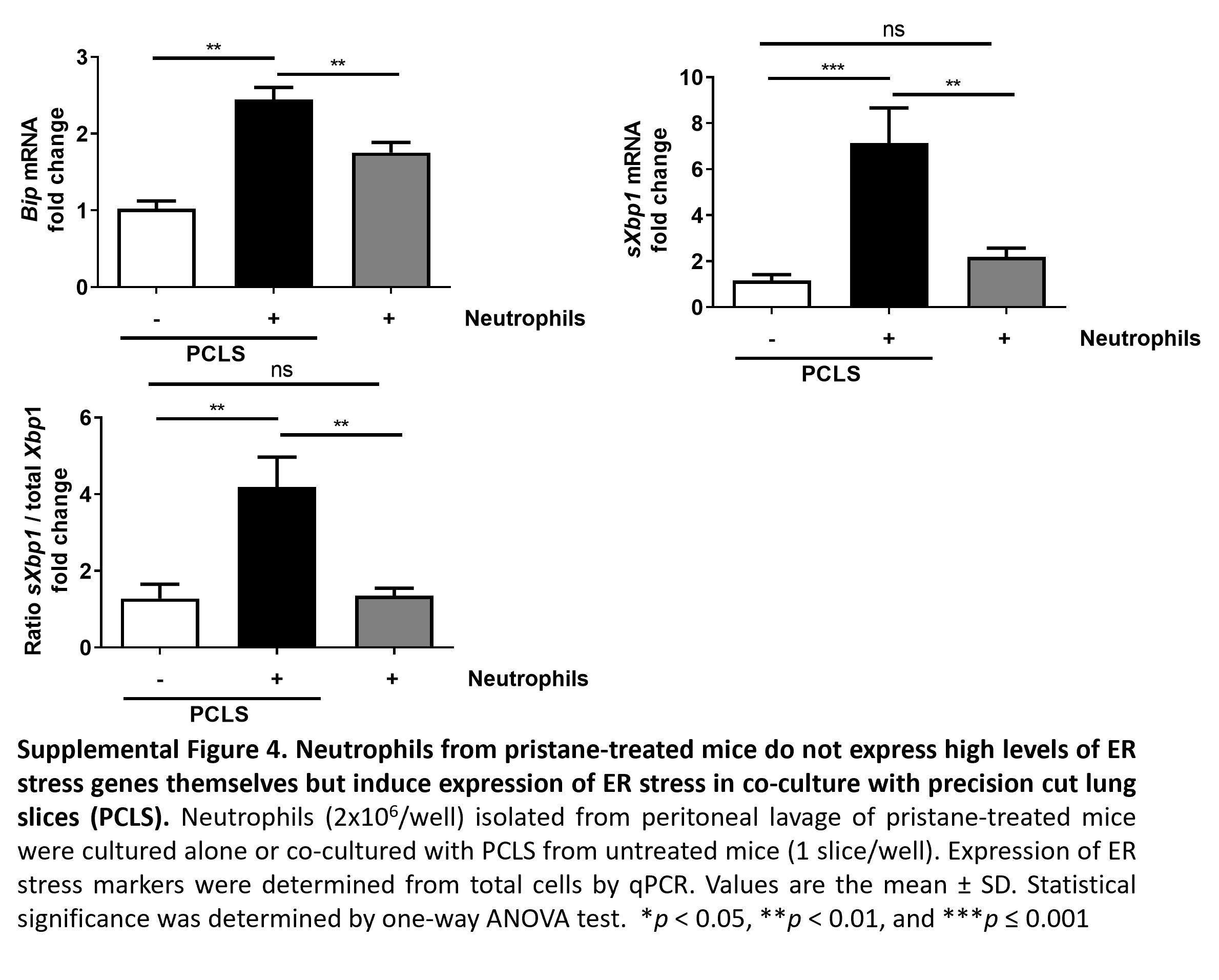

Supplement: Supplementary file 4 [file Image_4.jpg]

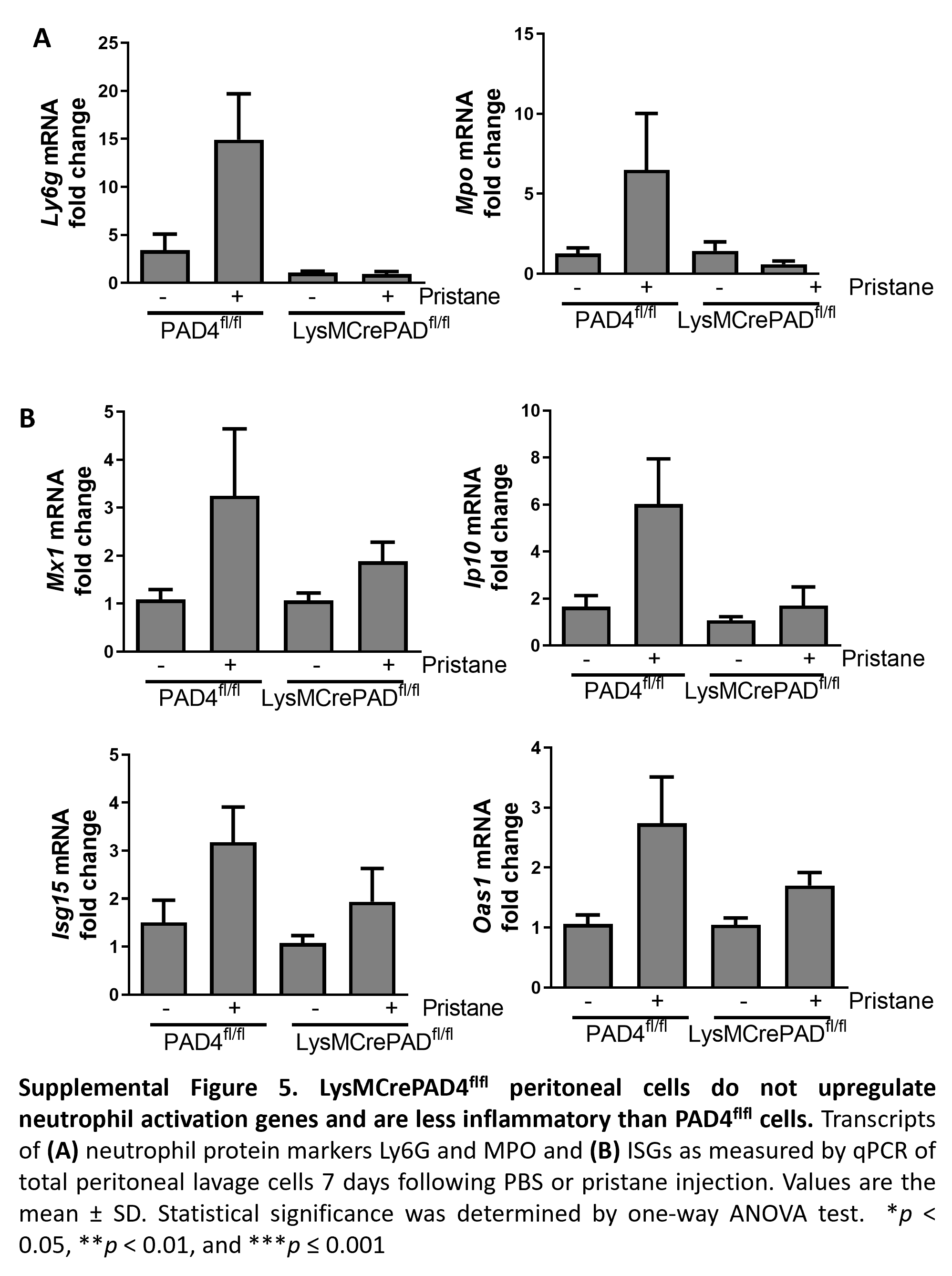

Supplement: Supplementary file 5 [file Image_5.jpg]

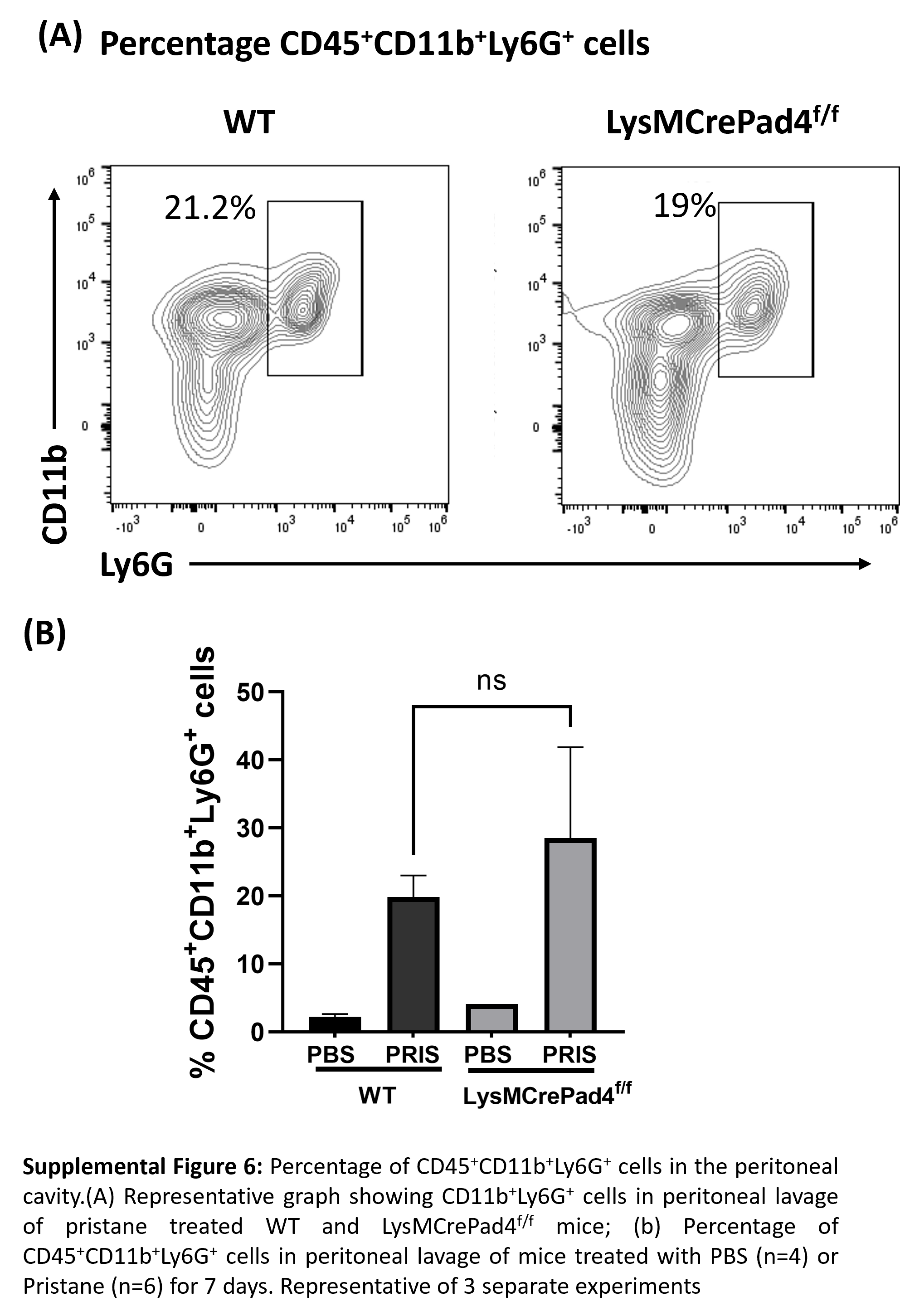

Supplement: Supplementary file 6 [file Image_6.jpg]
