# Supplementary material for: Neutrophils Contribute to ER Stress in Lung Epithelial Cells in the Pristane-Induced Diffuse Alveolar Hemorrhage Mouse Model
Source: Front Immunol. 2022 Feb 3;13:790043. doi: 10.3389/fimmu.2022.790043 (PMC8850275; doi:10.3389/fimmu.2022.790043)
Supplement: Supplementary file 7 [file Table_1.docx]

| **Murine primer sequences:** | | |
| --- | --- | --- |
| *Gapdh* | 5’-CATCAAGAAGGTGGTGAAGC-3’ | 5’-CCTGTTGCTGTAGCTGTATT-3’ |
| *Ddit3* | 5’-ACGGAAACAGAGTGGTCAGTGC-3’ | 5’-CAGGAGGTGATGCCCACTGTTC-3’ |
| *Hspa5* | 5’-CCTGCGTCGGTGTGTTCAAG-3’ | 5’-AAGGGTCATTCCAAGTGCG-3’ |
| *sXbp1* | 5’-GAGTCCGCAGCAGGTG-3’ | 5’-GTGTCAGAGTCCATGGGA-3’ |
| *Xbp1* | 5’-GTCCATGGGAAGATGTTCTGG-3’ | 5’-TGGCCGGGTCTGCTGAGTCCG-3’ |
| *Ip10* | 5’-GAGTCCGCAGCAGGTG-3’ | 5’-GTGTCAGAGTCCATGGGA-3’ |
| *Oas1* | 5’-GCCTGATCCCAGAATCTATGC-3’ | 5’-GAGCAACTCTAGGGCGTACTG-3’ |
| *Isg15* | 5’-GGTGTCCGTGACTAACTCCAT-3’ | 5’-CTGTACCACTAGCATCACTGTG-3’ |
| *Mx1* | 5’-GATCCGACTTCACTTCCAGATGG-3’ | 5’-CATCTCAGTGGTAGTCAACCC-3’ |
| *Ifnb* | 5’-CAGCTCCAAGAAAGGACGAAC-3’ | 5’-GGCAGTGTAACTCTTCTGCAT-3’ |
| *Elane* | 5’- GACATGACGAAGTTCCTGGCA-3’ | 5’-CCTTGGCAGACTATCCAGCC-3’ |
| *Mpo* | 5’- AGTTGTGCTGAGCTGTATGGA-3’ | 5’-CGGCTGCTTGAAGTAAAACAGG-3’ |
| **Human primer sequences:** | | |
| *18S* | 5’-GCTTAATTTGACTCAACACGGGA-3’ | 5’-AGCTATCAATCTGTCAATCCTGTC-3’ |
| *ATF4* | 5’-GTTCTCCAGCGACAAGGCTA-3’ | 5’-ATCCTGCTTGCTGTTGTTGG-3’ |
| *CHOP* | 5’-AGAACCAGGAAACGGAAACAGA-3’ | 5’-TCTCCTTCATGCGCTGCTTT-3’ |
| *IFI44L* | 5’-AGCCGTCAGGGATGTACTATAAC-3’ | 5’-AGGGAATCATTTGGCTCTGTAGA-3' |
| *IL16* | 5’-GCCGAAGACCCTTGGGTTAG-3’ | 5’-GCTGGCATTGGGCTGTAGA-3’ |
| *IL8* | 5’-AAATTTGGGGTGGAAAGGTT-3’ | 5’-TCCTGATTTCTGCAGCTCTGT-3’ |
| *IP10* | 5’-GTGGCATTCAAGGAGTACCTC-3’ | 5’-TGATGGCCTTCGATTCTGGATT-3’ |
| *ISG15* | 5’-ACTCATCTTTGCCAGTACAGGAG-3’ | 5’-CAGCATCTTCACCGTCAGGTC-3’ |
| *RSAD2* | 5’-TTGGACATTCTCGCTATCTCCT-3’ | 5’-AGTGCTTTGATCTGTTCCGTC-3’ |
| *sXBP1* | 5’-CTGAGTCCGAATCAGGTGCAG-3’ | 5’-ATCCATGGGGAGATGTTCTGG-3’ |
| *XBP1* | 5’-TGGCCGGGTCTGCTGAGTCCG-3’ | 5’-ATCCATGGGGAGATGTTCTGG-3’ |
